# Epidemiologic Characteristics of Patients Admitted to Emergency Department with Dermatological Complaints; a Retrospective Cross sectional Study

**Published:** 2019-08-19

**Authors:** Deniz Kilic, Ozlem Yigit, Taylan Kilic, Cagri Sefa Buyurgan, Ozlem Dicle

**Affiliations:** 1 Department of Emergency Medicine, Kepez State Hospital, Antalya, Turkey.; 2 Department of Emergency Medicine, Faculty of Medicine, Akdeniz University, Antalya, Turkey.; 3 Department of Emergency Medicine, Antalya Education and Research Hospital, Antalya, Turkey.; 4 Department of Emergency Medicine, Ömer Halisdemir University Education and Research Hospital, Nigde, Turkey.; 5 Department of Dermatology, Faculty of Medicine, Akdeniz University, Antalya, Turkey.

**Keywords:** Emergency medicine, urticaria, Referral and Consultation, Exanthema, Anaphylaxis

## Abstract

**Introduction:**

Dermatological diseases constitute 5-8% of all emergency department (ED) visits. However, little is known about these patients. The aim of this study is to determine the epidemiologic characteristics of patients admitted to ED with dermatological complaints.

**Methods:**

This is a retrospective cross-sectional study conducted in the ED of a university hospital. Patients over 18 years of age who presented to ED with the following complaints were included in the study: rash, pruritus, and edema sensation in the throat or shortness of breath due to an allergic reaction. Demographics, chief complaints, final diagnoses, triage categories, consultations and hospitalization rates were obtained through computerized database of the hospital.

**Results:**

859 patients were included in the final analysis. 511 (59.5%) patients were female and the mean age of patients was 39.03±15 years. The most common complaint and final diagnosis were skin rash with pruritus (50.9%) and urticaria with drug eruptions (84.5%), respectively. Two patients (0.2%) had an emergent triage category. 804 (93.6%) patients were discharged from ED, while 55 (6.4%) received consultations, resulting in 19 (34.5%) hospitalizations.

**Conclusion:**

Most of the patients admitted to ED with dermatological complaints are non-urgent and can be treated as outpatients. However, physicians should be alert for emergent causes, as well.

## Introduction

Although dermatology is often thought of as a non-acute and outpatient centered clinic, it has been reported that approximately 4-12% of all emergency department (ED) visits are due to skin complaints (1). Many of these skin lesions are caused by infections, irritants and allergens (2). Most of the dermatological lesions presenting to the EDs are neither serious nor life-threatening. However, patients usually seek immediate attention and keep on crowding the ED with non-urgent complaints. 

Dermatological emergencies involve less severe or life-threatening diseases such as infectious skin diseases (e.g. abscesses, cellulitis and necrotizing fasciitis), acute rashes, severe cutaneous adverse reactions (e.g. drug rash with eosinophilia and systemic symptoms (DRESS) syndrome, Stevens Johnson Syndrome (SJS), and toxic epidermal necrolysis (TEN)), erythroderma, vasculitis, flares of chronic inflammatory skin diseases, urticaria, and angio-edema (3). It is important for an emergency physician (EP) to recognize and treat dermatological emergencies, as some of these conditions can acutely evolve and become lethal if the diagnosis is not made early in the disease course and the appropriate treatment is not provided in time (4). Relieving the patient’s complaints, prescribing appropriate treatment, and recommending outpatient control visits to the dermatology clinics for nonurgent diseases are the other important responsibilities for the EPs. 

There are few studies documenting patients’ characteristics and ED consultations. Therefore, the aim of this study is to describe the epidemiological characteristics of the patients admitted to the ED with a dermatologic complaint. As a secondary aim, we sought to get epidemiological data about our population and use these data for planning the content of our emergency residency program about the most common dermatological diseases. 

## Methods

**Study design and setting**

This was a retrospective cross-sectional study conducted in the ED of a university hospital with an annual turnover of approximately 100,000 adult patients. The study was approved by the local ethical committee (code: 18/05/2016–288; From Akdeniz University 2012-KAEK-20). 

Patients who were over 18 years old and admitted to the ED with a dermatological complaint during a 6-month period were included in the study. Pruritus, rash, erythema, and swelling in the throat and dyspnea due to an allergic reaction were presumed to be the major complaints that could be seen in the ED. The resident EPs were asked which International Code of Disease-10 (ICD-10) codes they were using for recording the patients with dermatological complaints. The codes identified after this preliminary questionnaire were searched in the electronic database of the hospital. The identified ICD-10 codes were L29 (pruritus, unspecified), L50 (urticaria), L50.8 (other: chronic urticaria), L50.9 (urticaria, unspecified), R21 (rash and other nonspecific skin eruption), T78.2 (anaphylactic shock), T78.3 (angioneurotic edema), and T78.4 (other and unspecified allergy). Patients’ demographics and complaints, final diagnoses, triage categories, time of the ED admissions, consultations, discharge and hospitalization rates, and control visits to the outpatient dermatology clinics of the hospital after discharge from the ED were searched and recorded in the study form. 

***Participants***

Since ED admissions of the children were carried out in the pediatric ED of the hospital, patients under the age of 18 were excluded from the study. Patients who had revisited the ED within five days were also excluded due to their ongoing treatment. Also, when ICD-10 codes were asked from resident EPs, it was learned that they were using ICD-10 codes of L03 (cellulitis) and other infectious skin disorders in different manners. In case of a necessity to make a laboratory testing for differential diagnosis between infectious and noninfectious causes or to prescribe an antibiotic even for prophylactic purposes, an ICD code of an infectious cause must be registered into the electronic database of the patient to cover the health insurance payment system. The retrospective design of the study had made it difficult to differentiate between mannered and actual diagnoses. Therefore, cellulitis and other infectious skin disorders were not included in the study.

***Data collection***

The demographics of the patients, admission complaints, time of the ED admissions (hours in a day and month), triage categories, referral from other hospital, consultations, and discharge and hospitalization ratios were recorded in the study form. Revisit to the ED within five days after discharge and the control visits to the dermatology clinic were also recorded. Admission complaints were classified as; pruritus, rash, itchy erythematous skin rash, insect bite, and edema sensation in the throat and dyspnea due to an allergic reaction. ED admission time was classified as; 08:00-16:59 daytime, 17:00-23:59 evening, and 00:00-07:59 night. Triage categories were classified as green (non-urgent), yellow (urgent-delayed), and red (emergent). Due to the inability to differentiate between urticaria and drug eruptions, these final diagnoses were combined as a single diagnosis as urticaria and drug eruptions. Similarly, a combination was also made for angioedema and anaphylaxis, as well.

***Statistical analysis***

Data were analyzed with the IBM SPSS 20.0 statistical package program. Continuous data are presented as mean± standard deviation (SD), and categorical data as frequencies and percentages. Univariate analyses between two groups for categorical data were performed using the Chi-squared test. A two-sided p value of <0.05 was considered as statistically significant. 

## Results

***Baseline characteristics of studied patients***

**Figure 1:**
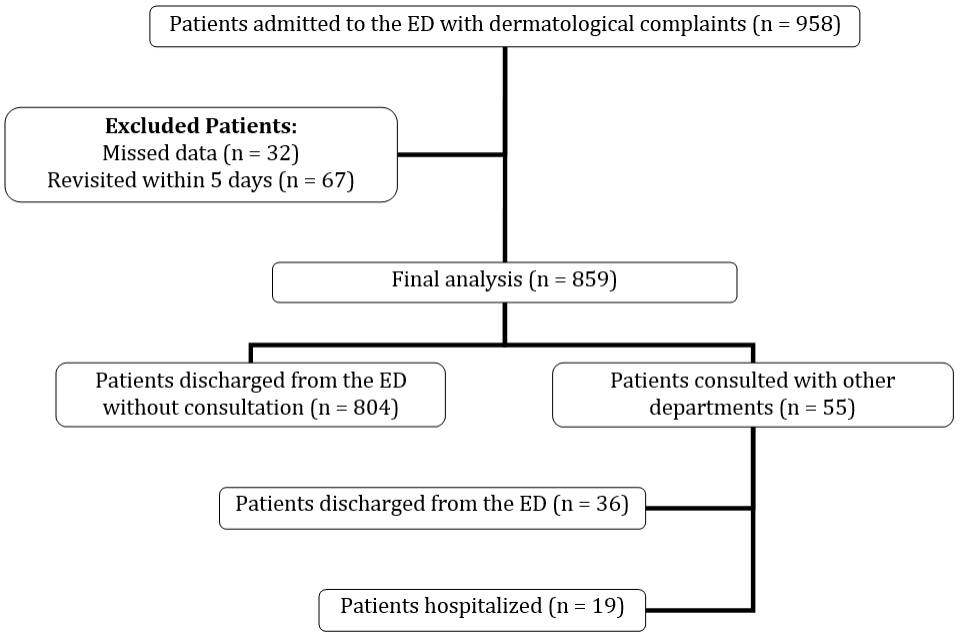
Patients flow chart. ED: emergency department, n: number

During the study period, 50622 patients were admitted to the ED. 958 (1.89%) of these patients had dermatologic complaints. After the exclusion of 99 patients, 859 patients were included in the final analysis. Patient flow-chart is depicted in Figure 1. The mean age was 39.03±15 (18-89) years and 511 (59.5%) patients were female. The most common dermatological complaint was erythematous skin rash with pruritus (%50.9). Also, most of the patients had presented to the ED between 08:00-16:59 hours in the daytime (45.9%) and were in a triage category of yellow (%98.8). Patients’ characteristics are depicted in Table 1. 

**Figure 2:**
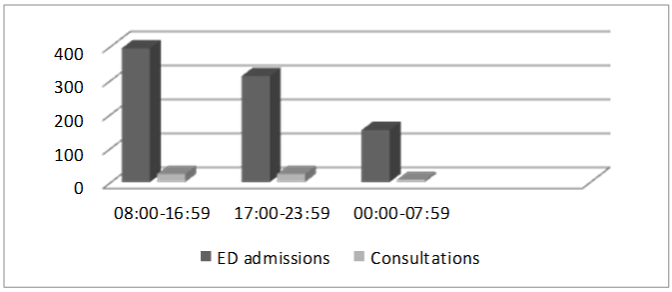
Distribution of emergency department (ED) admissions and consultations during a day

**Figure 3:**
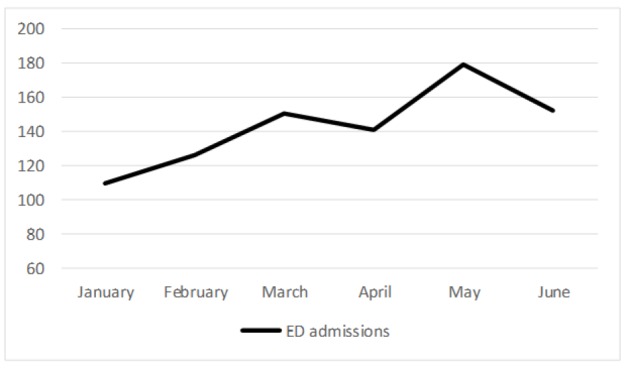
Number of the emergency department admissions of patients with dermatological complaint in months

The most common final diagnosis was urticaria and drug eruptions (84.5%). Most of the patients were admitted to the ED between 08:00-16:59 hours (%45.9) and, most of the consultations took place in 08:00-16:59 (%43) and 17:00-23:59 (%43) time intervals. Time distribution of ED admissions and consultation in a day is depicted in Figure 2. When looking at a monthly basis, most of the patients were admitted in hot months (May: 20.8%, June: 17.7%) (Figure 3).

While 804 (93.6%) patients were discharged with prescriptions to home without any consultations, 55 (6.4%) patients received consultations from other clinics (dermatology, allergy-immunology or ear-nose-throat departments). Of these, 19 patients (2.2% of the study population and 34.5% of the consulted patients) were hospitalized in the related clinics. 58 (6.8%) patients were referred from another hospital. These patients had statistically and significantly higher consultation (p=0.002) and hospitalization (p=0.034) rates than the others. 

***Outcomes***

It has been shown that, patients with the final diagnosis of angioedema and anaphylaxis have significantly higher consultation (p=0.025) and hospitalization (p=0.004) rates than the urticaria patients. Among the patients who were discharged from ED with prescription and instructions, only 122 (15.2%) patients visited the outpatient dermatology clinic of our hospital for control visits. The rate of control visits to the outpatient dermatology clinic was also significantly higher in patients who were consulted during the ED process (27.3% vs 15.2%, p=0.018).

A total of 55 patients had revisited the ED within five days after discharge from the ED. While 46 patients had only one revisit, seven patients had two, one patient had three and another patient had four revisits to the ED. There wasn’t any statistically significant difference between the consultation rates of the patients with revisits to the ED and the patients without revisits (9.1% and 6.2%, respectively, p=0.389). 

**Table 1:** Baseline characteristics of studied patients

**Characteristics**	**Number (%)**
**Age (years)**	
Mean ± SD	39.03±15.03
**Gender**	
Male	348 (40.5)
Female	511 (59.9)
**Admission complaints**	
Itchy and erythematous skin rash	437 (50.9)
Other rashes	145 (16.9)
Angioedema and/or difficulty in breathing	129 (15.0)
Insect bite	21 (2.4)
**Triage category**	
Non-urgent	9 (1.0)
Urgent	848 (98.8)
Emergent	2 (0.2)
**Time of admission (hours)**	
08:00-16:59	394 (45.9)
17:00-23:59	312 (36.3)
00:00-07:59	153 (17.8)
**Final diagnosis**	
Urticaria and drug eruptions	726 (%84.5)
Angioedema and anaphylaxis	124 (%14.4)
PUPPP	6 (%0.7)
Other (DRESS, SJS)	3 (%0.3)
Total	859 (%100)

PUPPP: pruritic urticarial papules and plaques of pregnancy, SJS: Stevens–Johnson syndrome, DRESS: drug rash with eosinophilia and systemic symptoms.

## Discussion

Our study shows that the most common dermatological complaint was erythematous skin rash with pruritus and the most common final diagnosis was urticaria. Patients presented to emergency department in day-time and many of them were not in a life-threatening situation or urgency. Also the rate of control visits to the outpatient dermatology clinic was very low. 

Urticaria-angioedema, SJS, TEN, necrotizing fasciitis, and erythroderma are the dermatologic emergencies that can be life-threatening (3). Other dermatologic complaints, which can be classified as non-urgent are frequently encountered in the ED. In different reports, the rate of dermatological complaints in all admissions to the ED was reported to be 2.5-8% (5-9). The ratio in our study was found to be 1.89% and it was lower than the previous reports. The infectious diseases, which were not included in our study, like cellulitis or others may be the cause of this lower rate. 

The predominance of women in our study was similar with the previous reports. We have found a male/female ratio of 0.68. This ratio was 0.85 and 0.71 in the studies by Rubegni et al. and Grillo et al., respectively (6, 7). In most other studies, this finding was explained by the greater concern among women over skin diseases. While the average age in our study was similar with a previous report from our country (10), it was younger than the other reports from Italy and Spain (7, 11). The reason for this difference may be that we have a younger population than Europe. In the previous studies the mean number of visits was significantly higher and more common during the summer months compared to other months (3, 8, 11). The visits were more frequent in May and June in our study; however, the difference was not statistically significant. 

Most of the patients were admitted to the ED between 8:00-16:59 in the daytime. Both family physicians’ primary care offices and dermatology clinics were open and available in this time frame. The question “Why people with non-urgent complaints visited the ED instead of getting an appointment from a specialist working at outpatient clinics?” used to be a curiosity and has been asked by several researchers in the past. As an answer to this question, a report from France has revealed the following results: even though all patients had known that their situation had not been an emergency, yet concern about pain, discomfort or the course of their diseases had made them visit the ED. They had thought that they could not get a scheduled outpatient clinic appointment within a short time and had not wanted to wait a long time. Patients with multiple and various complaints had not wanted to program a few different outpatient clinic appointments and had preferred to use the ED clinics, in their words ‘‘where everything is gathered in one place and the doctors work fast’' (12). Despite the high prevalence of dermatoses in our population, it is difficult to obtain a timely dermatology appointment in our country, the same as many other countries. The mean waiting time for an appointment to the outpatient dermatology clinic ranges between approximately one week and three months. At public hospitals, waiting times are significantly longer as higher percentage of the patients with limited or no health insurance get service from these hospitals. It was, also, shown that dermatological primary diagnosis was more likely in lower-income and underinsured groups (13). Underinsured patients face higher rejection rates and longer waiting times for an appointment at outpatient dermatology clinics. Thus, these patients may increase the inappropriate use of the EDs for non-urgent dermatologic complaints. Since our study was in a retrospective design, we were not able to evaluate the insurance and income status of the study population.

Another possible explanation for the inappropriate use of the ED by young people could be the precarious nature of their working hours, which would prevent them from seeking medical care during the working days (3, 14). In our study, 36.3% of patients were admitted to ED between 17:00-23:59 hours. The patient number decreased between 00:00-07:59 at night, as expected. It might be thought that only really urgent cases may present during these hours. However, on the contrary, the consultation and hospitalization rates did not get any higher in this time interval. The lower ratio of real emergencies (red triage category) in our study, also, supports the argument that the patients use the ED inappropriately. 

The hospitalization rate of our study was 2%. This result was similar with the previous reports (6, 7). The ratio of hospitalization for the patients who were consulted in the ED was 34.5%. In a previous report, only 18% of consulted patients were hospitalized and the majority (82%) of these consultations were non-urgent (15). In a study performed at a tertiary care center in India, Gupta et al. reported that 21 of 100 emergency outpatient consultations qualified as ‘true dermatologic emergencies’ (16). Longer waiting time for outpatient care was proposed as a reason for non-urgent consultations by the doctors consulting the patients. This was also a reason for revisits to the ED, so the EPs preferred to consult these patients to prevent revisits to the ED. The consultation rate for revisit patients in our study was not statistically different from the patients who did not revisit the ED. 

Patients with a final diagnosis of anaphylaxis and angioedema had significantly higher rates of consultation and hospitalization than those with a final diagnosis of urticaria. This was also pertinent to the patients who were referred from another hospital to our ED, as well. Since our clinic is a tertiary care facility, more complicated patients were referred to our hospital. This may explain the higher hospitalization rate in our study. 

As in the previous reports, the most frequent group of disease was urticaria and drug eruptions (5-10, 17, 18). The most significant difference in our study was the final diagnosis for the infectious diseases and dermatoses. We have excluded the skin infections from the study due to the reasons explained before. However, absence of any dermatitis or eczema in the final diagnoses was problematic. Since the study had a retrospective design, we selected the patients with the ICD-10 codes, which we identified with a preliminary questionnaire before the study. The lack of codes for dermatoses among the most frequently used ICD-10 codes might have led to the inability to distinguish these patients in the study. The absence of these final diagnoses might be due to diagnostic ICD-10 codes being recorded to the electronic database of the hospital were either a nonspecific complaint code or an incorrect infectious disease code. Therefore, either way, it is more likely for dermatoses to be excluded from the study population than these cases never visiting the ED.

Of all the patients who were discharged from ED with prescription and instructions, only a few patients visited the dermatology outpatient clinic for control examinations, and the remaining 84.8% were only seen by an EP. Dermatology is a specialty in its own right and it is unlikely that the EP will approach the patient from a dermatologist’s point of view. However, the EP is obliged to recognize and treat dermatological emergencies. In addition, it is also true that a substantial part of patients with non-urgent dermatological complaints visit the ED instead of outpatient clinics for various reasons. This is not only a problem in our country, but also in the health services of the entire world. A number of studies have been conducted in order to canalize non-emergent patients to the outpatient clinics rather than ED, and policies are being implemented according to the results. However, since the social norms are difficult to change, these policies will not show a positive impact in the near future. In this case, it is inevitable that the EP should have the knowledge to identify the most common dermatologic diseases other than the dermatological pathologies that should be urgent or emergent and manage their treatment. Therefore, identification of common dermatological complaints and related diagnoses and shaping the ED residency training program according to the results is essential. Taking into consideration the results of our study, it may be suggested that giving more emphasis to the curriculum of our country, especially those related to urticaria and drug eruption, would be beneficial both for the education of EPs and patient care. Unlike other studies, the fact that no dermatoses were found in our study suggests that physicians have difficulties in recognizing this diagnosis and complementary trainings may also be useful in these situations. Selecting common visual dermatological lesions and presenting them to the residents at the weekly clinical session for discussion of the diagnosis, treatment, and outcome may be a practical learning method. 

## Limitations

We have some limitations. Most importantly, this was a retrospective study and the data was gathered through the patients’ files, thus limiting its efficacy. Our study covers a period of six months. This makes it difficult to make clear a comment on the distribution of complaints and final diagnoses on a monthly and seasonal basis. This might, also, have created a bias for some diseases such as being in the favor of insect bites. Also, there were no clear explanations about drug eruptions in the patient files. Therefore, we combined two distinct final diagnoses of urticaria and drug eruption into a unique final diagnosis as urticaria and drug eruption. The same was also done for angioedema and anaphylaxis diagnoses. Patients with other dermatologic diagnoses recorded with different codes may have been excluded from the study, as the study population was generated through the complaints and the commonly used ICD-10 codes determined by common usage of the resident EPs before the study. Exclusion of the cellulitis and other infectious causes was, also, a major limitation. A prospective design and at least one year of follow up survey can overcome these limitations.

## Conclusion: 

Since most of the patients presenting to ED with dermatological complaints are non-urgent and can be treated as outpatients, EPs should have the knowledge to identify the most common dermatologic diseases other than the dermatological pathologies that should be urgent or emergent and manage their treatment. Therefore, identification of common dermatological complaints and related diagnoses and shaping the ED residency training program according to the results is essential.
